# Differentiating mindfulness-integrated cognitive behavior therapy and mindfulness-based cognitive therapy clinically: the *why*, *how,* and *what* of evidence-based practice

**DOI:** 10.3389/fpsyg.2024.1342592

**Published:** 2024-02-06

**Authors:** Sarah E. B. Francis, Frances Shawyer, Bruno A. Cayoun, Andrea Grabovac, Graham Meadows

**Affiliations:** ^1^Southern Synergy, Department of Psychiatry, School of Clinical Sciences at Monash Health, Monash University, Melbourne, VIC, Australia; ^2^Mindfulness-integrated Cognitive Behavior Therapy Institute, Hobart, TAS, Australia; ^3^Department of Psychiatry, University of British Columbia, Vancouver, BC, Canada; ^4^Mental Health Program, Monash Health, Melbourne, VIC, Australia; ^5^Melbourne School of Population and Global Health, University of Melbourne, Melbourne, VIC, Australia; ^6^School of Public Health and Preventive Medicine, Monash University, Melbourne, VIC, Australia; ^7^School of Primary and Allied Health Care, Monash University, Melbourne, VIC, Australia

**Keywords:** mindfulness-integrated cognitive behavior therapy, mindfulness-based cognitive therapy, mindfulness-based programs, mindfulness, depression, transdiagnostic

## Abstract

It is important to be able to differentiate mindfulness-based programs in terms of their model, therapeutic elements, and supporting evidence. This article compares mindfulness-based cognitive therapy (MBCT), developed for relapse prevention in depression, and mindfulness-integrated cognitive behavior therapy (MiCBT), developed for transdiagnostic applications, on: (1) origins, context and theoretical rationale (*why*), (2) program structure, practice and, professional training (*how*), and (3) evidence (*what*). While both approaches incorporate behavior change methods, MBCT encourages behavioral activation, whereas MiCBT includes various exposure procedures to reduce avoidance, including a protocol to practice equanimity during problematic interpersonal interactions, and a compassion training to prevent relapse. MBCT has a substantial research base, including multiple systematic reviews and meta-analyses. It is an endorsed preventative treatment for depressive relapse in several clinical guidelines, but its single disorder approach might be regarded as a limitation in many health service settings. MiCBT has a promising evidence base and potential to make a valuable contribution to psychological treatment through its transdiagnostic applicability but has not yet been considered in clinical guidelines. While greater attention to later stage dissemination and implementation research is recommended for MBCT, more high quality RCTs and systematic reviews are needed to develop the evidence base for MiCBT.

## Introduction

In the last 40 years, there has been an extraordinary take-up of mindfulness-based programs (MBPs) in both clinical and well-being contexts. Many different programs are now available (Creswell, 2017), and many of these have been based on, or adapted from, Mindfulness-Based Stress Reduction (MBSR) (Kabat-Zinn, 1990; Crane et al., 2017). This program, created initially to address chronic pain, was inspired by Asian meditation and yogic traditions including Zen and Vipassana practices to teach about the mind–body connection. MBSR utilizes mindfulness exercises, meditation and yoga and had a focus on regulating attention (Kabat-Zinn, 1982). Subsequently, key elements of MBSR were adapted to develop Mindfulness-Based Cognitive Therapy for Depression (MBCT; Segal et al., 2002, 2013), a group intervention to promote relapse prevention in recurrent depression based on the cognitive model of depressive relapse. In contrast to MBCT, Mindfulness-integrated Cognitive Behavior Therapy (MiCBT) was developed with transdiagnostic mental health applications in mind, piloted in psychiatric populations with both moderate and severe symptoms, and designed for both individual and group contexts (Cayoun, 2011, 2015; Cayoun et al., 2019). Because it is transdiagnostic, MiCBT can also address comorbidities (Barlow et al., 2004) and it is increasingly being used to promote well-being and personal growth in the general community (Cayoun, 2015).

The aim of this paper is to inform clinicians and researchers about the unique features of MiCBT and MBCT to enable appropriate referrals and assist with decisions to pursue professional training and research pathways. As noted by Kazdin (2016), with the large number of evidence-based treatments (EBTs) now available, “a key challenge is disseminating EBTs so they are used in clinical practice. Extending EBTs to practice has enormous challenges of its own beginning with what to train among the burgeoning list of treatments.” (p. 428). It has been suggested that examining the similarities and differences between MBPs will assist in refining understanding of their respective clinical applications (Chiesa and Malinowski, 2011). To this end, the objectives of this article are to: describe and compare the origins, theoretical models, and therapeutic elements of MiCBT and MBCT to promote a deep understanding of *why* and *how* they are applied to treat their target clinical populations; to consider *what* their corresponding evidence base is; then consider the implications of this comparative review for clinical applications.

## Origins and theory

The original naming of the three MBPs provides interesting insight into the respective developers’ initial conceptualizations. MBSR started out as “Stress Reduction and Relaxation Program” (Kabat-Zinn, 1982) as Kabat-Zinn wanted to introduce mindfulness and meditation practices to Western medical settings, and, to increase acceptability, intentionally did not explicitly emphasize their Buddhist roots. It wasn’t until the 1990s that the name “Mindfulness-Based Stress Reduction” was formally used for the program (Kabat-Zinn, 2011). Additionally, the word “stress” had already been popularized in the medical community, for whom the Buddhist use of the word “suffering” may have been less defined (Kabat-Zinn, 1990, p. 235). The original name for MBCT was ‘Attention Control Training’ (Teasdale et al., 1995) because learning to skillfully deploy one’s attention was seen as a central process to the prevention of depressive relapse (Dimidjian et al., 2010). MiCBT was initially implemented in 2001 as ‘Equanimity Training’ as an intervention for acute conditions and relapse prevention across disorders, because of the focus on developing equal interest, acceptance and non-reactivity towards body sensations regardless of their affective valence, especially during distressing experiences (Cayoun et al., 2004).

Mindfulness was defined by Kabat-Zinn as “paying attention in a particular way; on purpose, in the present moment, and non-judgmentally” (Kabat-Zinn, 1994, p. 4). This definition was adopted in the first edition of the MBCT manual (Segal et al., 2002, p. 121) but later amended to include awareness: “the awareness that emerges from paying attention, on purpose, in the present moment, and nonjudgmentally” (Segal et al., 2013, p. 132). This wording aligns with a consensus-developed definition published in 2004: “We see mindfulness as a process of regulating attention in order to bring a quality of nonelaborative awareness to current experience and the quality of relating to one’s experience within an orientation of curiosity experiential openness and acceptance. We further see mindfulness as a process of gaining insight into the nature of one’s mind and the adoption of a de-centered perspective (Safran and Segal, 1990) on thoughts and feelings” (Bishop et al., 2004, p. 230). In MiCBT, the definition of mindfulness overlapped, but includes the central role of equanimity, the non-reactive component of mindfulness, as understood in from its Buddhist roots. Accordingly, mindfulness is considered to be achieved through the development of experiential awareness and equanimity “in such a way that we can perceive our experiences, understand them and respond to them without needing to react in order to change them. This includes experiences that are taking place within the mind and body” (Cayoun, 2015).

### MiCBT origins and theory

Following over a decade of conceptualization and development beginning in 1989, MiCBT (Cayoun, 2011; Cayoun et al., 2019) emerged in Australia independently, but at a similar time to the publication of the first MBCT text (Segal et al., 2002). The initial name of Equanimity Training (2001–2003) became Mindfulness-based CBT (MCBT) from 2003–2006 then finalized as MiCBT from 2006 to avoid confusion with MBCT (Cayoun, 2011). MiCBT arose from Cayoun’s interest in bringing Vipassana meditation into a scientific and clinical context to assist with transcending the sources of unnecessary suffering (Cayoun et al., 2019). MiCBT was developed as a manualized transdiagnostic psychological treatment based on an integration of mindfulness meditations (in the Burmese Vipassana lineage of Ledi Sayadaw and U Ba Khin, as taught by Satya Narayan Goenka; Hart, 1987) with cognitive and behavioral therapy principles and methods. The first formal pilot evaluation commenced in 2003 in a psychiatric hospital setting (Cayoun et al., 2004). Further piloting and refinement took place until 2010, culminating in the first published MiCBT clinical text and manualized protocol (Cayoun, 2011). The MiCBT program, while retaining its structure and content, has been revised from 8- to 10-weeks duration (Cayoun, 2011; Cayoun et al., 2019). For further details on the history of MiCBT, see Cayoun et al. (2019).

MiCBT incorporates the four types of mindfulness trainings originally taught in Buddhist teaching on “The Establishment of Mindfulness,” the *Satipatthana Sutta* (Ñāṇamoli and Bodhi, 2005). These include mindfulness of the body (posture and movement), body sensations (interoception and hedonic tone), mental states (e.g., moods; aversive, craving, and mindful states), and mental qualities (presence of various types of thoughts). MiCBT emphasizes the importance of developing non-reactive interoceptive awareness through mindfulness of body sensations. Interoception, the ability to sense internal signals from the body, allows the perception of hedonic tone or valence (feeling pleasant, unpleasant and neither pleasant nor unpleasant body sensations) associated with emotional experiences (Barrett, 2006; Pollatos and Schandry, 2008). As training in mindfulness of body sensations improves the capacity for interoception, one becomes increasingly emotionally aware (Gibson, 2019). It is now well documented that emotional reactivity, conscious or not, is dependent on interoceptive experience (Menon and Uddin, 2010; Uddin, 2015; Zamariola et al., 2019). Numerous studies have demonstrated that interoception is impaired to varying degrees in mental health disorders (Garfinkel and Critchley, 2013; Khalsa et al., 2018; Zamariola et al., 2019) and impaired interoceptive awareness is a major contributor to emotional reactivity (Rae et al., 2019).

Reactivity to emotional valence is expressed through craving for pleasant sensory experiences and avoidance of unpleasant ones. According to the teaching of the Goenka lineage, difficulty in managing emotional reactivity is maintained by lack of awareness that this reactive process is continually taking place (Hart, 1987). The development of wise, compassionate equanimity to all arising internal phenomena is seen as a skillful way to navigate suffering (Hart, 1987; Batchelor, 2018). In the psychological literature, equanimity has been defined as “an even-minded mental state or dispositional tendency toward all experiences or objects, regardless of their affective valence (pleasant, unpleasant or neutral) or source” (Desbordes et al., 2015, p. 4). Equanimity enables the hedonic experience to be decoupled from action choices (Hadash et al., 2016). Consequently, equanimity towards interoceptive signals facilitates emotion regulation (Füstös et al., 2013; Hadash et al., 2016).

**Figure 1:**
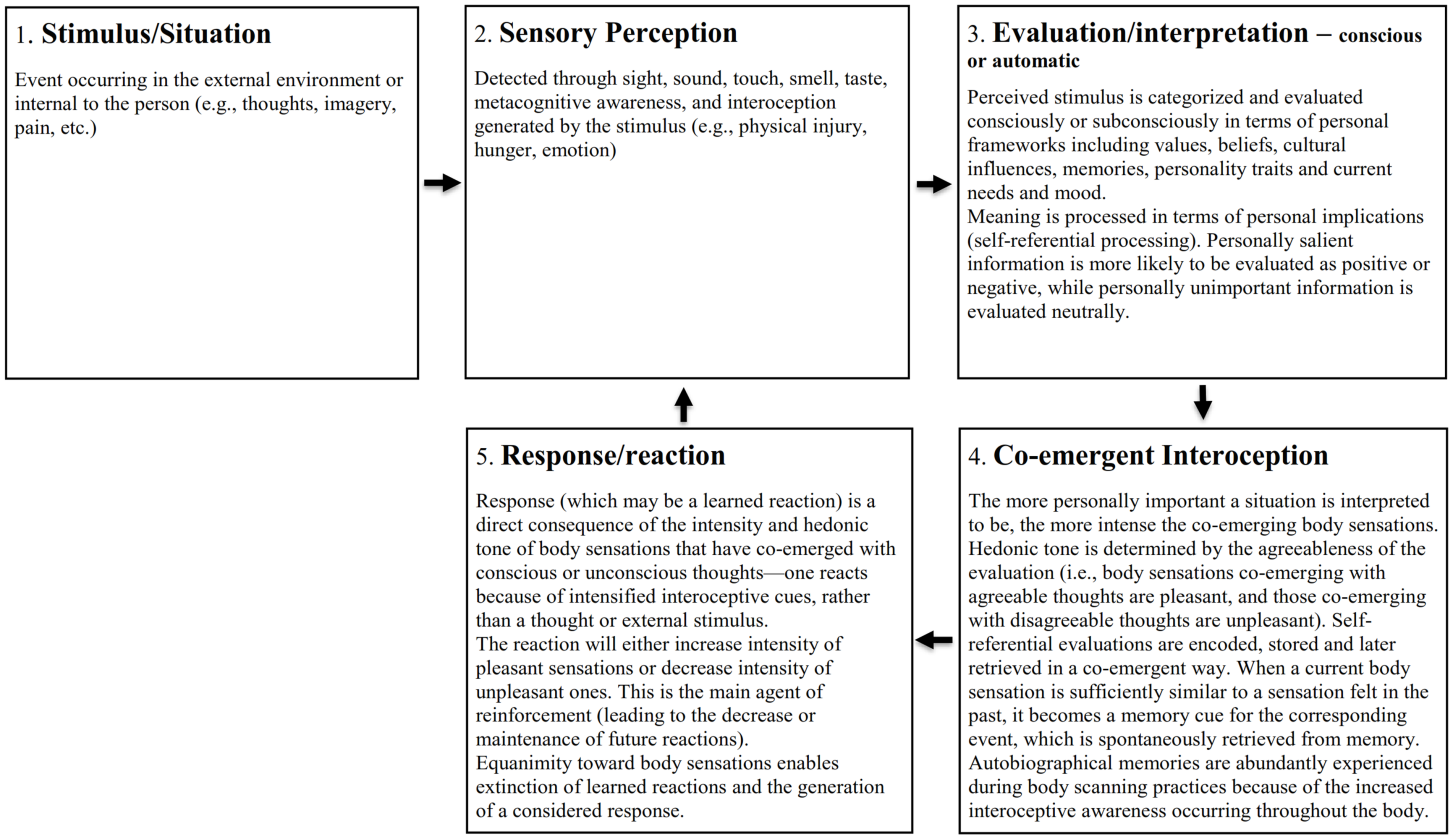
The co-emergence model of reinforcement in equilibrium state (Adapted from Cayoun, 2011).

MiCBT incorporates exposure and other behavior change techniques from CBT which have been demonstrated to be effective strategies in treating various types of anxiety and depressive disorders, and other related conditions (Barlow et al., 2004). In MiCBT, behavior change strategies incorporate operant learning principles and are guided by the co-emergence model of reinforcement (Figure 1), which underpins the program’s theoretical framework and rationale. The model describes the relationship between a stimulus event, the way this is processed cognitively, the co-arising sensations in the body and the subsequent reaction or response. This model is derived from Theravada Buddhist teachings on “the five aggregates” of information processing (Bodhi, 2003) and the neurophenomenology of behavior (see Cayoun and Shires, 2020).

Figure 1 shows the model components in their equilibrium state. In summary, a stimulus is perceived *(Sensory Perception)* through the senses (sight, touch, smell, taste, sound) or through awareness of thoughts or interoception, and is immediately consciously or subconsciously interpreted and evaluated *(Evaluation).* The stimulus is evaluated in terms of its personal significance, determined by cognitive factors such as values, beliefs, memories, personality traits, current mood, perceived needs, and culture. This causes *Co-emerging Interoception* of pleasant, unpleasant, or neutral body sensations to arise synchronously with evaluative thinking (see Cayoun and Shires, 2020, for discussion of neural correlates). If the co-emerging body sensations are sufficiently intense, a *Reaction*, (the next component of the model), is likely to occur. Reinforcement takes place if the reaction leads to a decrease in unpleasant body sensations or an increase in pleasant ones.

Hence, the model proposes that the reinforcement of behavior depends on the response to co-emerging interoception, rather than a response to the stimulus. This is theoretically consistent with learning theory (Mowrer and Klein, 2000) but differs from Skinner’s standard operant conditioning model, which assumes that “the behavior operates upon the environment to generate consequences” (Skinner, 1953, p. 65).

When well-being is disturbed, a disequilibrium state occurs, in which the Evaluation and Reaction functions predominate, causing the Sensory Perception and Co-emerging Interoception functions to be depleted. When the predominance of self-referential evaluation and reactivity becomes habitual, the disequilibrium state is established, facilitating the persistence of mental health conditions and the strengthening of reactive personality traits (Cayoun et al., 2019). The ability to pay attention to the precise nature of arising body sensations without reacting to them deepens perception of current internal experience and develops equanimity. As equanimity develops, reactivity decreases (Hadash et al., 2016) and a state of equilibrium between components can be restored. Consequently, reappraisal of beliefs about experience becomes possible. This principle is applied across the four stages of MiCBT (discussed below), where interoceptive desensitization is central to behavior change. In summary, co-emergence theory posits that cognition and interoception co-emerge bidirectionally, and being overly judgmental and reactive reduces sensory perception and interoceptive awareness, causing disequilibrium in the system, which may become learned over time and promote mental health disorders. Since interoceptive deficit and emotional hyper-and hypo-reactivity are common in emotional disorders, the development of equanimity towards interoceptive cues has transdiagnostic applicability. The co-emergence model of reinforcement is described in further detail elsewhere (Cayoun, 2011; Cayoun et al., 2019; Cayoun and Shires, 2020).

### MBCT origins and theory

MBCT is focused on mental health (depression in the first iteration) and, as such, targets specific conditions, departing from the transdiagnostic approach of MBSR in which the term ‘stress’ subsumes a multitude of conditions including stress, pain and medical illness (Kabat-Zinn, 2011). MBCT was designed as an eight-week manualized group training program for prevention of depressive relapse and includes mindfulness exercises and aspects of cognitive therapy (CT) for depression (Segal et al., 2002, 2013). The mindfulness elements of MBCT are mostly adapted from MBSR which in turn, and like MiCBT, are informed by the Satipatthana meditation methods (Kabat-Zinn, 1982). The one exception to this is the 3-min breathing space exercise which was introduced in MBCT as a mini-practice to be utilized at any point throughout the day (Teasdale et al., 2014).

MBCT developed out of a quest to create a maintenance version of CT for patients in remission, building on acute-phase CT which had been shown to reduce rates of depressive relapse (Segal et al., 2002). The developers were attracted to the affective and cognitive strategies in MBSR (Dimidjian et al., 2010), in particular, the ability of the program to focus on “learning to deploy one’s attention in specific and intentional ways, a skill that seemed highly relevant to helping patients notice early warning signs of depression” (Dimidjian et al., 2010, p. 309). Furthermore, the developers were impressed by the ability of MBSR participants to develop awareness of their own thought and thinking processes, decentering from thoughts to facilitate clear-sightedness (Segal et al., 2013).

After initially unsuccessfully trialing a 20-min mindfulness practice adapted from MBSR as part of Attentional Control Training (Teasdale et al., 1995), the developers observed that MBSR also included asking participants to have a welcoming attitude towards all experience, thus extending their earlier conceptualization of decentering to encompass “even the most intense negative experiences utilizing decentering more widely and deeply than we did” (Segal et al., 2013, p. 59). This represented a significant shift from the traditional CT method of reducing difficult emotions by working to modify thoughts and solve problems to an approach that focused on changing the attitude to thoughts and all experiences. Several other techniques from CT and CBT were incorporated into MBCT including activity scheduling, thought monitoring, differentiating thoughts from facts, promoting cognitive rehearsal, becoming aware of dysfunctional attitudes, and developing an early warning system and relapse prevention plan (Segal et al., 2002).

MBCT is primarily described in terms of a cognitive theoretical perspective rather than the Buddhist-based origins of MBSR (Segal et al., 2013). The MBCT program is based on an enhancement of cognitive theory using the information-processing theory of depressive relapse (Teasdale et al., 1995) and integrating mindfulness. The Interactive Cognitive Subsystems model suggested by Barnard and Teasdale (1991) proposes a ‘central engine’ in which sensory information is either coded in a propositional (factual) way or in an implicational (self-referential) way, in terms of its personal importance. Implicational thinking in which self-oriented ruminative thinking may create negative self-schemas then prolongs depressive symptoms (Nolen-Hoeksema and Morrow, 1991; Teasdale et al., 1995).

The cognitive model of depression typically posits that symptoms of depression develop from irrational assumptions or beliefs (implicational thinking) resulting in automatic negative thoughts that are associated with unpleasant emotions (Hawton et al., 1989). Incidents can trigger a dysfunctional belief that sets in motion a vicious cycle in which the more one becomes depressed, the more one engages with and believes the depressive thoughts, establishing schematic thought patterns. Accordingly, relapse prevention was initially proposed to be accomplished by modifying the dysfunctional schema (Hawton et al., 1989). However, among populations receiving CT for depression, rates of depressive relapse continued to be high, between 30 and 40% (Lin et al., 1998; Van Weel-Baumgarten et al., 2000) and new strategies to decrease this were sought. The role of a more metacognitive mode of processing information described as distancing or ‘decentering’ from thoughts, was proposed as a possible mediator of cognitive therapy’s effects (Ingram and Hollon, 1986). It was this quest to develop a therapy that focused on cultivating decentering that led to the development of MBCT, as outlined above.

The core skills taught in MBCT are awareness of thoughts and feelings, control of attention towards thinking patterns, decentering from thoughts to prevent being caught up in them, and the ability to experience present reality as it is (Teasdale et al., 2002). This more accepting and acknowledging stance towards thoughts and the maintenance of attention (e.g., on breath) provides alternatives to elaborative, ruminating thinking. There is evidence to suggest that MBCT improves attentional capacity (Jha et al., 2007; Semple, 2010; Grossman, 2011). MBCT describes attachment and aversion as important drivers of thinking patterns (Segal et al., 2002) and in the revised protocol examines ‘feeling tone’, described as “each experience we have …automatically evokes a feeling that is pleasant, unpleasant or neutral” (Segal et al., 2013, p. 217). Feeling tone is described as subtle, and often below the conscious threshold of awareness, but key therapeutically, as pleasant feeling tone is habitually reacted to with attachment, unpleasant feeling tone with aversion, and neutral feeling tone with disconnection and boredom. MBCT focuses on aversion as it “is at the root of all the states of mind that underlie relapse in depression” (Segal et al., 2013, p. 217); the focus on aversion to unpleasant feeling tone is specifically applied to the consequences of challenging thoughts and rumination.

## Program structure and practices

### MiCBT program structure and practices

MiCBT is delivered in four stages. Stage 1 (personal stage) focuses on experiential-awareness and regulation of attention and emotions through mindfulness meditation; Stage 2 (exposure stage) is a behavior-regulation stage integrating mindfulness meditation with exposure methods; Stage 3 (interpersonal stage) focuses on developing mindfulness-based interpersonal skills using the tools developed in the previous two stages; Stage 4 (empathic stage) is a transpersonal regulation stage to help prevent relapse by developing a sense of interconnectedness through integrating mindfulness, compassion and ethics for the maintenance of well-being.

Practices in MiCBT, whether delivered individually or in groups, are scheduled in a specific sequence to develop metacognitive insight, interoceptive awareness, and equanimity. These skills are then used to assist with understanding the relationship between unhelpful thinking, co-emerging body sensations and consequent affective reactivity. Reflection on, and engagement with, the practices of both meditation and behavioral tasks are facilitated using Socratic questioning. Prior to the first session, a standard clinical assessment is done, commitment to self-care practices is established, targeted problems are identified, and success indicators are proposed for each issue. A week-by-week summary of the program is provided in Supplementary Table S1. To begin with, patients practice daily progressive muscle relaxation to establish a regular self-care practice and to bring awareness to body, posture, movements, and tensions throughout the day and to anchor their attention in the present. In the second session, mindfulness of breath meditation is taught to develop metacognitive awareness and insight. The practice requires sustaining attention on the sensations associated with the breath at the entrance of the nostrils, without controlling the breath in any way. Participants sit upright and are instructed to focus on the breath, noticing thoughts arising without engaging or identifying with them, calmly shifting attention back to the breath when their mind wanders (Cayoun, 2011).

From session three onwards, a series of body-scanning techniques in the ancient Theravadin tradition of U Ba Khin and Goenka are introduced, and each is practiced for a week. The purpose of these methods is to progressively increase interoceptive awareness and equanimity toward the feeling tone of body sensations. Body scanning in MiCBT is done sitting up straight and systematically surveying the body, initially vertically (from head to toe and toe to head) and then transversally (scanning from surface to internal sensations). Participants are instructed to remain alert and equanimous while experiencing body sensations as they arise, without imagining anything, visualizing body parts, seeking relaxation, or otherwise affecting the actual experience. Apart from during the first week, the experience of relaxation may occur as a by-product of equanimity. It is expected that meditators will have a range of pleasant, neutral, and unpleasant experiences as they continue with their practice (Grabovac, 2015). From session 4, exposure techniques are introduced. The first involves applying equanimity during stressful situations in daily life using the Mindfulness-based Interoceptive Exposure Task (MIET). Patients are taught to monitor arising body sensations for short periods at a time while remaining equanimous to neutralize their reactivity. The Mindfulness-based Interoceptive Signature Scale (Cayoun, 2011) is used to record their interoceptive experiences pre- and post-exposure, describing sensations in terms of their physical characteristics of mass, temperature, movement and density. Thus, the MIET cultivates both equanimity and the ability to directly experience the transient and impersonal nature of sensations, resulting in an experience-derived reappraisal of distress.

In sessions five and six, behavior change is accomplished using equanimity-based imaginal and *in-vivo* exposure techniques. In sessions seven and eight, these techniques are applied to assist in developing interpersonal insights and ‘mindful assertiveness’. In sessions nine and ten, compassion for oneself and others is introduced through loving kindness meditation. Compassion is defined as “the will to extend oneself for the purpose of minimizing one’s own or another’s suffering.” (Cayoun, 2017, p. 177). The practice involves pairing the pleasant flow of subtle body sensations, frequently present during body scanning at this stage of training, with compassionate thoughts towards self and others. These include positive affirmations such as “may I be peaceful,” “may I be kind to myself” and “may I share my compassion with all beings.” The pairing of pleasant body sensations with positive affirmations is in contrast with the sense of hopelessness associated with negative affirmations such as “I am useless” and “no one cares about me” which co-emerge with unpleasant sensations that can maintain depression.

Compassion training provides an ideal context for introducing ethical behavior in daily life to prevent harm to oneself and others. MiCBT explicitly teaches harm minimization within the framework of compassion training on the basis that it is not possible to be compassionate while willingly causing harm (Cayoun, 2017). This is consistent with the traditional Theravada view of ethics and compassion in which “the inclination toward kindness and compassion for others also contributes to increasingly wholesome ethical standards, since if compassion is a primary emotion in the relationship, lying, stealing, harming, or taking sexual advantage of another would be anathema” (Amaro, 2015, p. 72). Patients are invited to adopt five basic ethical challenges for a week as a behavioral experiment, and to notice any effects on their sense of well-being at an interoceptive level. In other words, they are asked to observe how it feels (internal sensations) if they behave unethically and compare this to how it feels when they deliberately inhibit the behavior. In line with the view of Kabat-Zinn (2006), ethics are understood as necessary for the development of accurate and effective mindfulness practice.

### MBCT program structure and practices

As noted earlier, except for the 3-min breathing space, the mindfulness practices used in MBCT are derived from MBSR, with the rationale in MBCT linking to the cognitive model as described above (see also Supplementary Table S1). The first half of the MBCT program has its focus on mindfulness practices contextualized in terms of working “skillfully with the thoughts, emotions, and bodily sensations and emotions that create vulnerability to depressive relapse” (Dimidjian et al., 2010, p. 319). The second half of the program focuses on preventing depressive relapse, building on acceptance towards unpleasant sensations, the early detection of negative thinking, and decentering from ruminative thought patterns to reduce habitual reactivity. Skillful action for taking care of oneself is developed as an alternative response to habitual reactions to negative thoughts and low mood and the work culminates in a relapse prevention action plan. Each class is thematically organized and taught via the practices and subsequent group inquiry processes whereby learnings at each session are reflected upon and consolidated.

The first mindfulness exercise in session 1 is mindful eating (the raisin exercise), which is followed by a body scan (Segal et al., 2013), taught lying down or seated. Attention is first directed to the abdomen, then to the toes and moved up through the whole body while imagining directing the breath into and out from the different body parts, with instructions such as, “When you are ready, on an inbreath, feel or imagine the breath entering the lungs, and then passing down into the abdomen, into the left leg, the left foot, and out to the toes of the left foot. Then on the outbreath, feel or imagine the breath coming all the way back up, out of the foot, into the leg, up through the abdomen, chest, and out through the nose.” (Segal et al., 2013, pp. 122–123). Instructions include becoming aware of intense sensations and to “‘breathe in’ to them…. and… have a sense of their letting go, or releasing, on the outbreath” (Segal et al., 2013, p. 123). In session 2, a mindfulness of the breath exercise is introduced in which attention is brought to breathing at the abdominal area. A pleasant events calendar is also presented in session 2 to bring awareness to how pleasant feeling tone is experienced. In session 3, the sitting meditation incorporates mindfulness of the breath with awareness of sensations throughout the body. Mindful stretching is also introduced in session 3 as well as an unpleasant events calendar and a 3-min breathing space. The 3-min breathing space is offered as way to pause during stressful experiences during the day and turn towards the intensity of what is being experienced, instead of automatically reacting. Participants are invited to recognize and acknowledge their experience in the moment, then to bring focused attention to the breath, and finally to “expand attention to include a sense of the breath and body as a whole” (Segal et al., 2013, p. 196). Mindful stretching, mindful seeing and mindful hearing exercises are included in sessions 3 and 4. In session 4, the sitting meditation is mindfulness of sounds and thoughts, and mindful walking is also introduced. Additionally, in session 4 participants are introduced to the Automatic Thoughts Questionnaire as psychoeducation on further dis-identifying from some of the common cognitive distortions that occur in depression, by seeing them as symptoms of the illness, underscoring that thoughts are “just thoughts” and not facts.

In session 5, the working with difficulty meditation is taught in which awareness is brought to the area of strongest physical sensations evoked in the body when thinking of a difficulty, with an attitude of acceptance and openness to the sensations, intentionally letting go of any bracing or resistance. This practice is introduced as a way of letting go of aversion and cultivating an attitude of acceptance and friendliness. There is also a full day of mindfulness practice between Session 6 and 7, during which sitting and movement-based practices are alternated. The mountain meditation is introduced; this practice uses the image of a mountain to assist with feeling grounded regardless of the ‘weather systems’ that might come and go in the form of painful sensations or difficult thoughts.

In Session 6 and 7 the focus is on the relationship between thoughts, actions, and mood: in session 6, seeing moods as transitory and, in session 7, acting skillfully. MBCT invites a choice of mindfulness practices and after all have been learned, participants self-select which mediation practice to use for daily home practice. Throughout the program, the 3-min breathing space is used to apply skills learned during formal mindfulness practice into daily life, especially during times of stress or difficulty. The breathing space is used as the first step when starting to feel depressed. It is followed by asking oneself “How can I best take care of myself right now?,” to inform next actions, for example, an activity that provides a sense of pleasure or accomplishment.

In MBCT and MBSR, ethics and compassion are described as being included implicitly (Kabat-Zinn, 2011; Segal et al., 2013; Crane et al., 2017) through teachers embodying and role-modelling an ethical and kind stance as part of the program (Segal et al., 2013; Crane et al., 2017).

The possible conditions to which specific MBPs may be applicable are proposed in Table 1. Supplementary Table S1 provides a week-by-week overview of the program themes and practices used in MBCT and MiCBT.

**Table 1:** Possible applications of selected mindfulness-based programs.

**Current health status – mental/physical**	**MBSR**	**MBCT**	**MiCBT**
Well – seeking well-being/existential issues	**✓**		**✓**
Well – in remission looking to prevent depressive relapse		**✓**	**✓**
Stress related issues with physical illness	**✓**		**✓**
Stress related issues – life circumstances	**✓**	**✓**	**✓**
Mild to moderate depression – acute	**✓**	**✓**	**✓**
Mild to moderate depression – relapsed		**✓**	**✓**
Anxiety	**✓**	✓*	**✓**
Anxiety with other co-morbidities including depression		✓*	**✓**
Chronic pain	**✓**	✓*	**✓**
Bipolar depression – adjunctive		✓*	**✓**
Attention deficit hyperactivity disorder – MBCT adjunctive		✓*	**✓**
Post-traumatic stress disorder		✓*	**✓**
Anger management		✓*	**✓**
Obsessive compulsive disorder		✓*	**✓**

* With adaptations to original program.

## Professional training

Professional training for both MBCT and MiCBT is rigorous and requires a substantial commitment of time and effort. Both require trainees to have clinical mental health qualification and strongly recommend ongoing development of their own meditation practice, including 5- and 7-day retreats.

### MiCBT professional training

Prospective MiCBT therapists re required to complete two online courses for accreditation: a foundational course for self-implementation of MiCBT, followed by an applied course for delivery of MiCBT with patients. Each of these courses contains 10 teaching sessions of 2–3 h delivered over 12 weeks. Further supervision sessions and demonstration of the necessary competence is required to obtain MiCBT certification, available after at least 1 year of delivering MiCBT in a clinical practice setting at which time they may be added to the MiCBT Institute’s international registry of certified therapists.^1^ The above training program is offered through a centralized training institute located in Australia,^2^ with various international MiCBT Institute Chapters. Assessment criteria are outlined in the MiCBT Certification Process.^3^

### MBCT professional training

The MBCT training program typically takes place over a 1 to 3-year period, with facilitators expected to achieve competency across all six domains of the Mindfulness-Based Interventions – Teaching Assessment Criteria (MBI-TAC) (Crane et al., 2013). MBCT training organizations are widespread – available in at least 11 countries.^4^ There is variation in training delivery and some training programs have moved online. There is increasing flexibility as to how training requirements are met. Training generally includes experiencing an MBCT group as a participant-observer, attending teacher training (online or traditionally as an in-person 5-day retreat) and delivering MBCT classes in a supervised capacity. There is no central certification process for MBCT, however MBCT therapists can apply to be assessed for listing on the ACCESS MBCT Register.^5^ Assessment criteria are outlined in the MBCT Training Pathway.^6^

## Evidence base

### MiCBT evidence base

Randomized controlled trials (RCTs) have provided evidence that MiCBT is efficacious in treating anxiety and depression in pregnant women (Yazdanimehr et al., 2016), in women with multiple sclerosis (Bahrani et al., 2017), and in men and women with type 2 diabetes (Sohrabi et al., 2022). Other studies report MiCBT to be efficacious in managing and reducing chronic pain (Cayoun et al., 2020) and increasing pain self-efficacy in patients with breast cancer (Mozafari-Motlagh et al., 2019), reducing anxiety, depression and fatigue while improving sleep quality and hope in patients with multiple sclerosis (Pouyanfard et al., 2020), reducing body image-related distress and improving emotion regulation in cancer survivors following mastectomy (Rasouli et al., 2023), reducing substance addiction compared to established treatment (Wickham, 2013), and reducing sports-anxiety and pessimism and increasing flow and adherence in competitive athletes (Scott-Hamilton et al., 2016). MiCBT was piloted in a heterogenous sample in a psychology clinic context and was equally effective when delivered in groups and individual therapy sessions (Roubos et al., 2011; cited in Cayoun et al., 2019). A recent RCT demonstrated that MiCBT was significantly more effective compared to a treatment-as-usual waitlist control group in decreasing clinical symptoms of anxiety, depression and stress and improving flourishing in a transdiagnostic group (Francis et al., 2022). Another recent study demonstrated that gene expression (IncRNA) improved in women with perinatal depression who received training in MiCBT (Wang et al., 2021). A randomized controlled study comparing the effectiveness of four treatments for obsessive compulsive disorder, MiCBT, standard CBT, Acceptance and Commitment Therapy (ACT) and Metacognitive Therapy (MT), found that while all treatments were similarly effective at mitigating symptoms from pre- to post-treatment, only the participants in the MiCBT and ACT groups maintained their gains at follow-up (Derakhtkar et al., 2022). While the evidence base for MiCBT is growing, a search through PubMed and PsycINFO in August 2023 using the search terms systematic review, meta-analysis, MiCBT, and Mindfulness-integrated cognitive behavior therapy did not identify any published systematic reviews or meta-analyses.

Clinical guidelines in Australia, the United Kingdom (UK), the United States (USA), and Canada do not mention MiCBT although CBT and MBCT are mentioned and Australian guidelines noted that there were similar effect sizes for CBT, MBCT, and IPT (Biesheuvel-Leliefeld et al., 2015). Guidelines for the treatment of anxiety proposed ‘mindfulness-based therapies’ as structured interventions for treatment-resistant panic, social, and generalized anxiety disorders (Andrews et al., 2018). These guidelines recommend treatments that have evidence-based treatment manuals and can be modified to an individual patient while attending to the therapeutic relationship.

### MBCT evidence base

MBCT is currently the most widely researched MBP for mental health, and for depression in particular (Galante et al., 2013; Goldberg et al., 2019). In a search of PsychINFO, MEDLINE, Google Scholar in to August 2023 for systematic reviews and meta-analyses using key words MBCT, mindfulness-based cognitive therapy, systematic review, meta-analysis, recurrent, depression, and depressive relapse, we found one review (Coelho et al., 2007) and four meta-analyses (Piet and Hougaard, 2011; Kuyken et al., 2016; Goldberg et al., 2019; McCartney et al., 2020) that looked specifically at the efficacy of MBCT on the prevention of depressive relapse. These found advantages for its use particularly over treatment as usual, with the meta-analyses reporting moderate effect sizes. An additional systematic review looked at the management of depression with MBCT more broadly (Musa et al., 2020) and found that MBCT improved mindfulness and reduced relapse rates as well as depressive symptoms. In addition to reductions in depressive symptoms, meta-analyses from Zhang et al. (2022) and Tseng et al. (2023) also found reductions in suicidal ideation in depressed patients with large and small effect sizes, respectively. Although initial studies of MBCT suggested there may be an increased risk of relapse following MBCT for people who had experienced fewer than three episodes of depression (Teasdale et al., 2000; Helen Ma and Teasdale, 2004), a subgroup meta-analysis by McCartney et al. (2020), reported comparable results between those who had experienced more than three episodes and those who had experienced fewer than three episodes. Similarly, while the same earlier papers recommended the use of MBCT only in patients in remission from depressive episodes (Teasdale et al., 2000; Helen Ma and Teasdale, 2004), there is now an evidence base for the use of MBCT in treating current depression (Lenz et al., 2016; Goldberg et al., 2019; Liu et al., 2019; Thimm and Johnsen, 2020).

The use of MBCT with other conditions has also been studied including anxiety (Evans et al., 2008; Kim et al., 2009; Ghahari et al., 2020), bipolar disorder (Lovas and Schuman-Olivier, 2018; Xuan et al., 2020), vascular disease (Abbott et al., 2014), breast cancer (Chang et al., 2023), and chronic health conditions (Hazlett-Stevens et al., 2019; Pei et al., 2021). Meta-analyses examining the use of MBCT in populations living with bipolar disorder (Xuan et al., 2020) and chronic pain (Pei et al., 2021) found reductions in depression in between-group comparisons with moderate effect sizes. In contrast to Chang et al. (2023) meta-analysis however, Pei et al. (2021) paper did not find any statistically significant improvements in measures of pain. Though two meta-analyses reported reduction in anxiety symptoms with MBCT (Ghahari et al., 2020; Xuan et al., 2020), results varied for between-group analyses: Xuan et al. (2020) meta-analysis found no significant effects, as opposed to Ghahari et al. (2020) meta-analysis, which noted significant decreases with moderate effect sizes. A study on the implementation of MBCT in the U.K. health system suggests that MBCT has been offered to patients with a variety of other conditions including anxiety and chronic pain/fatigue for some time (Crane and Kuyken, 2013). However, guidelines for adapting MBCT (amongst other MBPs) for other conditions have been published only recently (Loucks et al., 2022).

In Australia, MBCT was endorsed by the Royal Australian and New Zealand College of Psychiatrists (RANZCP) as a preventive treatment during the maintenance phase of recurrent depression episodes (Malhi et al., 2021). In the United Kingdom, the National Collaborating Centre for Mental Health for the National Institute for Health and Care Excellence found that MBCT was recommended for individuals with a history of three or more depression episodes, who are currently stable but remain susceptible to relapse despite antidepressant medication, or who choose to discontinue antidepressant use (Royal College of Psychiatrists, 2023). In the United States, in addition to being recommended for depressive relapse prevention, MBCT was also recommended as an initial standalone depression treatment for adults, in contrast to cognitive-behavioral therapy which was recommended for use in conjunction with antidepressant medication for initial treatment (American Psychological Association, 2019). Similarly, Canada endorsed MBCT, positioning it as a second-line adjunctive treatment supported by Level 2 Evidence for acute depression, and as a first-line maintenance therapy backed by Level 1 Evidence (Parikh et al., 2016). While lacking a formal recommendation for anxiety treatment in Canada, MBCT was acknowledged as a promising approach for addressing anxiety and related disorders (Katzman et al., 2014).

## Summary and clinical considerations

MiCBT and MBCT share a broadly similar approach to mental health by incorporating mindfulness with cognitive and behavioral therapies. They are each based on clear and coherent theoretical models that are conveyed throughout the programs and justify the use of practices and actions relating to the target populations. Nevertheless, the purpose, models and practices have significant differences. While MiCBT is designed to be a transdiagnostic program for acute and chronic mental health disorders, and can address comorbid symptoms, MBCT is designed for patients in remission from multiple depressive episodes, a well population with vulnerability to relapse. While MiCBT applies highly specific mindfulness practices to address the processes that precipitate and perpetuate a broad range of psychological conditions, MBCT focuses on the flexible application of a broad array of practices to one specific psychological condition, especially depression.

Co-emergence theory underpins MiCBT with initial focus on metacognitive awareness using mindfulness of the breath then a strong emphasis on interoceptive awareness and equanimity towards body sensations, using progressively refined body scanning methods to address emotional reactivity. Reactivity to unpleasant sensations that co-emerge with dysfunctional thinking patterns is an important phenomenon because it is a ‘common factor’ contributing to psychological processing across a range of mental health disorders (e.g., in depressive or anxiolytic thinking). MiCBT integrates interoceptive awareness and equanimity with Learning Theory. Applied practices target avoidance, including in interpersonal situations. MiCBT also cultivates the benefits of ethics and compassion and their relapse-prevention effects.

Cognitive theory underpins MBCT with emphasis on decentering from depression-related thoughts and feelings and averting escalating negative thinking to prevent depressive relapse. From session 6, participants choose their own selection of practices and are encouraged to embed mindfulness into their daily life. MBCT proposes that it is the regularity of practice that is critical, not which specific practice that is used as all the practices have the same end – to cultivate the “being” mode and develop the capacity to decentre from rumination to enable a wiser response too low mood.

Thus, MiCBT and MBCT are distinct treatment modalities and so require their own specific professional training. Clinically, the selection between MiCBT and MBCT may be influenced not only by diagnosis, but also by the severity of psychological symptoms and the capacity of patients to engage with the exposure and compassion methods used in MiCBT. Some clients with a significant trauma background may experience distress from extensive body scanning and skillful guidance from therapists is required. It is also possible that compassion exercises will trigger threat or grief for some people, such as those with underlying depression and adverse backgrounds (Gilbert et al., 2011; Steindl et al., 2022). Indeed, one of the reasons the developers of MBCT avoided including explicit compassion exercises such as loving kindness practices was due to the risk of triggering underlying vulnerabilities in people who struggle with depression (Segal et al., 2013, see pp. 138–139). However, in MiCBT, working with underlying vulnerabilities is central to the program, including addressing avoidance patterns associated with past trauma. Therapists are well trained to assess patients for the possibility of such experiences, prepare them for treatment and provide support as required, for example recommending individual rather than group sessions in some circumstances and pacing practice progression more slowly according to response (for details, see Cayoun et al., 2019).

In MBCT, the mindfulness exercises are introduced in a gentle and inviting manner, always deferring to a patient’s perceived capacity to do each exercise. The variety of ways that mindfulness is introduced allows for choice of exercises and with the aim of supporting long-term practice (Segal et al., 2013). This invitational stance may make it more acceptable to those who initially struggle to engage in treatments that involve more challenging exercises. Thus, it is possible that MBCT may provide a solid foundation of mindfulness before the potentially more challenging content of MiCBT. While the delivery of MiCBT is also gentle and accepting, the program is more prescriptive in terms of type and ordering of exercises. Symptoms such as avoidant or compulsive behaviors may be better addressed with the desensitization methods of MiCBT, which applies interoceptive awareness and exposure methods to address conditions such as panic disorder, obsessive compulsive disorder and post-traumatic stress disorder. As different emotional disorders may present with differing levels of sensitivity to interoceptive cues (e.g., panic disorder) the addition of equanimity to treatment can enable the management of avoidant behavior. The addition of ethics and compassion (with the loving kindness meditation) can assist with relapse prevention, preventing harm to oneself or to others. For either therapy, good client preparation before undertaking the program is essential so that potential risks and benefits are clearly understood.

Potential iatrogenic effects of MBPs have attracted increased attention since the major studies of MBCT were published. In developing non-judgmental awareness, both interventions invite participants to pay attention to unpleasant internal experiences occurring during mindfulness practice and there is a complex relationship between this process and adverse effects (Farias et al., 2020; Aizik-Reebs et al., 2021; Binda et al., 2022). A recent RCT comparing MiCBT with a wait-list control that included an examination of adverse effects did not elicit reports of any serious adverse effects (Francis et al., 2022). Mediation analysis suggested that equanimity was the most influential mediator of distress reduction and may be a protective factor against potential adverse effects. However, the current literature would not yet support a systemic comparison between the two interventions on negative effects (Farias et al., 2020). This is an area that could benefit from more precise attention in the future though the applications of measures such as the Meditation Experiences Interview (MedEx-I) (Britton et al., 2021).

The main limitation for MBCT is its single condition model - primarily major depression but also when adapted to other conditions. While single disorder MBPs will continue to uniquely meet the needs of specific populations, there is a recognized need for transdiagnostic treatment methods to extend the range of treatment possibilities and take account of comorbidities (Crane and Kuyken, 2013). For instance, using a transdiagnostic treatment protocol instead of multiple single disorder protocols can be a more resource-efficient way of treating many commonly occurring mental health conditions. In clinical settings where MBPs are delivered in groups, group recruitment can be achieved more readily, allowing more individuals to be served and wait times for treatment reduced. In addition, there are cost and time savings in terms of training clinicians in only one transdiagnostic treatment protocol, rather than in multiple single disorder protocols. Moreover, a transdiagnostic approach has the important clinical advantage of addressing comorbidity effectively.

The main limitations for MiCBT are its evidence base, which may be regarded as promising rather than established, and that it has not yet been considered in clinical guidelines. While greater attention to later stage dissemination and implementation research is recommended for single disorder protocols such as MBCT (Dimidjian et al., 2010; Goldberg et al., 2019), more quality RCTs and systematic reviews are needed to develop the evidence base for transdiagnostic MBPs such as MiCBT. Nevertheless, given the potential role for transdiagnostic MBPs, we propose that MiCBT makes a unique contribution to CBT and mindfulness-based therapies. The addition of the transdiagnostic applicability of co-emergence theory, which places importance on equanimity toward interoceptive cues in addition to metacognitive awareness, provides a strong rationale for the suite of practices and techniques in MiCBT.

## Author contributions

SF: Writing – original draft, Writing – review & editing. FS: Supervision, Writing – original draft, Writing – review & editing. BC: Writing – original draft, Writing – review & editing. AG: Writing – original draft, Writing – review & editing. GM: Supervision, Writing – original draft.

## Footnotes

^1^https://www.mindfulness.net.au/for-community/find-a-therapist/

^2^https://www.mindfulness.net.au

^3^https://www.mindfulness.net.au/training/micbt-professional-development-certification/

^4^https://www.accessmbct.com/mbct-training-organisations/

^5^https://www.accessmbct.com/

^6^https://www.oxfordmindfulness.org/wp-content/uploads/2018/02/MBCT-Training-Pathway.pdf
