# Supplementary material for: Differentiating mindfulness-integrated cognitive behavior therapy and mindfulness-based cognitive therapy clinically: the why, how, and what of evidence-based practice
Source: Front Psychol. 2024 Feb 6;15:1342592. doi: 10.3389/fpsyg.2024.1342592 (PMC10880191; doi:10.3389/fpsyg.2024.1342592)
Supplement: Supplementary file 1 [file Table_1.pdf]

Supplementary Table 1.

Summary of program themes and practices for MBCT and MiCBT

Week	MBCT Program (Segal et al., 2013) Recommended practice time is 45 mins for six out of seven days each week			MiCBT Program (Cayoun et al., 2019) Recommended practice time is 30 mins twice a day		
	**Theme**	**Group Work**	**Home Practice**	**Theme**	**Group Work**	**Home Practice**
**1**	Explain the structure of each session including: practice session; home practice; group work; overview of next learning topic<br><br>Automatic pilot - understanding the difference between being on "automatic pilot" or a "doing mode" of ruminative thinking, which is a common feature of depression. Focus on intentionally paying mindful attention	Eating meditation - raisin exercise<br><br>Body scan exercise<br><br>Dyad discussion on dealing with obstacles to practice	Body scan<br><br>Mindfulness of routine activity<br><br>Mindful eating – at least one meal and throughout each day Complete practice record forms	Explain the structure of each session including: practice session; home practice; group work; overview of next learning topic<br><br>Introduction to MiCBT - four stages; goal setting; gaining commitment to daily practice	Stage 1 - Progressive muscle relaxation<br><br>Mindfulness of body	Progressive muscle relaxation twice a day with audio instructions<br><br>Awareness of body movements and posture in day-to-day activities<br><br>Complete practice record forms<br><br>Baseline measurements for assessing outcome (e.g. DASS 21; MSES-R; SWLS)

**2**	Another way of knowing: living in our heads - knowing through thinking as opposed to knowing by experiencing; using mindfulness of the body to learn direct experience	Body scan meditation<br><br>Thoughts and feelings exercise<br><br>Set up pleasant experiences calendar<br><br>Sitting practice - 10 min	Body scan 6 out of 7 days<br><br>Mindfulness of breath (10 min.) - 6 out of 7 days<br><br>Pleasant experiences calendar (one example daily)<br><br>Mindfulness of a routine activity<br><br>Complete practice record forms	Developing attention-regulation skills and metacognitive awareness	Mindfulness of breath<br><br>Nature of thoughts – external and internal stimuli; frequency, recency, and co-emergence effects	Mindfulness of breath - 30 mins twice a day with audio instructions<br><br>Awareness of breath and types of thoughts during the day<br><br>Attention regulation (practise of “Right Effort”) during the day<br><br>Complete practice record forms

**3**	Gathering the scattered mind: understanding the distracted mind; using the breath and body to be present; moving from doing to being mode	Seeing or hearing exercise - 5-minute<br><br>Sitting meditation - awareness of breath and body and how to respond to intense physical sensations<br><br>3-minute breathing space<br><br>Mindful stretching<br><br>Set up unpleasant experiences calendar	Stretch and breath meditation - days 1,3,5<br><br>Mindful movement meditation - days 2,4,6<br><br>Unpleasant experiences calendar (daily, using a variety of experiences)<br><br>3-minute breathing space – 3x daily<br><br>Complete practice record forms	Mechanisms of emotional reactivity in terms of the co-emergence model; interoceptive awareness and equanimity; increasing mindfulness of body sensations	Part by part body scanning - scanning the surface of the body from head to toe<br><br>Explaining the co-emergence model using the Diary of Reactive Habits	Body scanning – part by part, 30 mins twice daily with audio instructions<br><br>Use the Diary of Reactive Habits to reflect on a recent event<br><br>Fill out Interoceptive Awareness Indicator<br><br>Complete practice record forms

**4**	Recognizing aversion: observing the mind's tendency for rumination<br><br>Worry and mind wandering as escape	5-minute seeing or hearing exercise<br><br>Sitting meditation<br><br>Poem – Wild Geese<br><br>The territory of depression – Automatic Thoughts Questionnaire<br><br>3-minute breathing space<br><br>Mindful walking	Sitting meditation-mindfulness of sounds and thoughts (37 min.) - 6 out of 7 days<br><br>3-minute breathing space – regular (three times daily)<br><br>3-minute breathing space - responsive (whenever unpleasant feelings)<br><br>Complete practice record forms	Developing skill and understanding including practicing without audio instructions; adjusting pace of scanning	Part by part body scanning (no audio instructions and without moving) with commitment to remaining equanimous; acquiring insight by observing and accepting the transient and non-personal nature of sensations<br><br>Observing the impermanence of thoughts and sensations and learning not to identify with experiences - letting go of attachment to "I" or "mine"	Body scanning – part by part, 30 mins twice a day in silence (no audio instructions) while maintaining immobility and developing equanimity<br><br>Applied mindfulness practice – awareness of body sensations with equanimity during the day and pre and post sleep<br><br>Practicing applied equanimity using the Mindfulness-based Interoceptive Signature Scale (MISS)<br><br>Complete practice record forms

**5**	Allowing/letting be: intentionally allowing all experience, including turning towards the difficult	Sitting meditation – awareness of breath and body; noticing effects of difficulty on body and reactions to it<br><br>Breathing space<br><br>Poem – The Guest House	Working with difficulty meditation – days 1, 3, 5<br><br>Sitting with silence (unguided practice) – day 2, 4, 6<br><br>3-minute breathing space - regular (three times daily)<br><br>3-minute breathing space - responsive (whenever unpleasant feelings)<br><br>Complete practice record forms	Integrating mindfulness to external situations and overcoming avoidance	Stage 2 - Integration of mindfulness and behavior methods<br><br>Symmetrical body scanning (surveying both sides of the body together while maintaining immobility)<br><br>Immobility during scanning tasks from here on<br><br>Learning to notice very subtle sensations as indicators of distress arising<br><br>Addressing avoidance by selecting items to work on using Subjective Units of Distress Scale (SUDS) and bipolar exposure	Body scanning – symmetrical scanning 30 mins twice a day with audio instructions<br><br>Exposure tasks - using imaginal interoceptive and in-vivo exteroceptive exposure to address avoided situations<br><br>Complete practice record forms

**6**	Thought are not facts: relating differently to thoughts<br><br>Seeing the relationship between negative thoughts and moods	Siting meditation – focus on thoughts as mental events<br><br>Moods, thoughts and alternative viewpoints exercise<br><br>Use of breathing space as first step before taking wider view of thoughts<br><br>Early warning signs of depression - relapse signature	Choose from guided meditations – minimum 40 mins a day<br><br>3-minute breathing space - regular (three times daily)<br><br>3-minute breathing space - responsive (whenever unpleasant feelings)<br><br>Working wisely with depression and unhappiness worksheet<br><br>Complete practice record forms	Generalizing self-confidence and self-efficacy - advanced scanning and consolidating exposure skills	Partial sweeping body scanning (passing interoceptive attention in a continuous manner over large sections of the body)<br><br>Understanding avoidance and its gradual assimilation into the self-concept<br><br>Reviewing progress using distress relief on SUDS items<br><br>Understanding how perceptions in the present are determined by past experiences ("causes and conditions")	Body scanning – partial sweeping, 30 mins twice a day with audio instructions<br><br>Continuing to use exposure tasks to address most pervasively avoided situations<br><br>Complete practice record forms

**7**	How best can I take care of myself: skilful action for self-care	Sitting meditation - awareness of breath and body<br><br>Links between mood and activity exercise<br><br>Activity scheduling for low mood<br><br>3-minute breathing space as first step before mindful action<br><br>Identifying actions to deal with threat of relapse<br><br>Generate list of pleasure and mastery activities<br><br>3-minute breathing space or mindful walking	Select practice of choice to use regularly each day<br><br>Action plan for relapse prevention<br><br>3-minute breathing space - regular (three times daily)<br><br>3-minute breathing space - responsive plus action (whenever unpleasant thoughts or feelings)<br><br>Complete practice record forms	Developing interpersonal insight and addressing conflicts<br>Understanding “experiential ownership”	Stage 3 - Continued development of insight and equanimity while noticing subtle sensations as potential onset of emotion<br><br>Sweeping *en masse* body scanning (passing interoceptive attention in a continuous manner over the entire surface of the body, in a single vertical flow of attention)<br><br>Exploring the possibility of blissful experience to be noticed with equanimity, without attachment or fear of the experience<br><br>Learning to apply equanimity towards others’ reactivity	Body scanning – sweeping "*en masse*" 30 mins twice a day with audio instructions<br>Interoceptive and exteroceptive exposure to discomfort in interpersonal situations<br><br>Experiential ownership exercise<br><br>Complete practice record forms

**8**	Maintaining and extending new learning: planning for maintaining mindful way of being	Body scan<br><br>Review whole course, including relapse prevention action plan<br><br>Personal reflections<br><br>Keeping up momentum<br><br>Concluding meditation		Mindful communication skills – using exposure to address challenging interpersonal situations with equanimity and kind assertiveness	Transversal body scanning (passing attention part by part, transversally through the body interior, from front to back and back to front)<br><br>Assertive communication - using awareness and equanimity to gain clarity in conflicting situations while expressing through seven statements methodically arranged	Body scanning – transversal scanning 30 mins twice a day with audio instructions<br><br>Practice mindful assertiveness with experiential ownership<br>Complete practice record forms

**9**		Stage 4 - Empathic stage: preventing relapse by cultivating compassion and connectedness without others through ethical living - five ethical challenges	Sweeping *in depth* body scanning (passing interoceptive attention in a continuous manner internally and externally, in a single vertical flow of attention)<br><br>Feeling connected internally - realising that wholesome thoughts produce pleasant sensations - accepting self and others as they are<br><br>Understanding the relationship between ethical behaviour and compassion - five ethical challenges set for the week (preventing harmful speech, taking what is not given, taking lives, inappropriate sexual actions, and intoxication)	Body scanning – sweeping “in depth” (internally) 30 mins twice daily in silence, (without audio instructions)<br><br>Loving kindness meditation with audio instructions following internal sweeping (“in depth”)<br><br>Ethical challenges task as a “behavioral experiment” for one week<br><br>Complete practice record forms

Loving kindness meditation - positive affirmation-based practice acting as counterconditioning by pairing free flow of pleasant body sensations with previously aversive stimuli

**10**	Maintaining and cultivating well-being	Review of skills learned and review of goals<br><br>Importance of maintaining daily practice<br><br>Maintenance practice (45 min daily in silence, comprising 10 min of mindfulness of breath, 25 min of sweeping in depth, and 10 min of loving-kindness meditation)<br><br>Review of the program and measures of overall outcomes

Note. The MBCT Program also includes a ‘Day of mindfulness’ between weeks 6 and 7, in which 6.5 hours is devoted to sitting meditation, mindful stretching, body scan, mindful eating, walking meditation, mountain meditation, review of experience and discussion.
